# Docosahexaenoic Acid-Derived Neuroprotectin D1 Induces Neuronal Survival via Secretase- and PPARγ-Mediated Mechanisms in Alzheimer's Disease Models

**DOI:** 10.1371/journal.pone.0015816

**Published:** 2011-01-05

**Authors:** Yuhai Zhao, Frederic Calon, Carl Julien, Jeremy W. Winkler, Nicos A. Petasis, Walter J. Lukiw, Nicolas G. Bazan

**Affiliations:** 1 Neuroscience Center of Excellence, School of Medicine, Louisiana State University Health Sciences Center, New Orleans, Louisiana, United States of America; 2 Molecular Endocrinology and Oncology Research Centre, Centre Hospitalier de l'Université Laval Research Centre (CHUL), Quebec, Canada; 3 Department of Chemistry, Loker Hydrocarbon Research Institute, University of Southern California, Los Angeles, California, United States of America; Case Western Reserve University, United States of America

## Abstract

Neuroprotectin D1 (NPD1) is a stereoselective mediator derived from the omega-3 essential fatty acid docosahexaenoic acid (DHA) with potent inflammatory resolving and neuroprotective bioactivity. NPD1 reduces Aβ42 peptide release from aging human brain cells and is severely depleted in Alzheimer's disease (AD) brain. Here we further characterize the mechanism of NPD1's neurogenic actions using 3xTg-AD mouse models and human neuronal-glial (HNG) cells in primary culture, either challenged with Aβ42 oligomeric peptide, or transfected with beta amyloid precursor protein (βAPP)_sw_ (Swedish double mutation APP695_sw_, K595N-M596L). We also show that NPD1 downregulates Aβ42-triggered expression of the pro-inflammatory enzyme cyclooxygenase-2 (COX-2) and of B-94 (a TNF-α-inducible pro-inflammatory element) and apoptosis in HNG cells. Moreover, NPD1 suppresses Aβ42 peptide shedding by down-regulating β-secretase-1 (BACE1) while activating the α-secretase ADAM10 and up-regulating sAPPα, thus shifting the cleavage of βAPP holoenzyme from an amyloidogenic into the non-amyloidogenic pathway. Use of the thiazolidinedione peroxisome proliferator-activated receptor gamma (PPARγ) agonist rosiglitazone, the irreversible PPARγ antagonist GW9662, and overexpressing PPARγ suggests that the NPD1-mediated down-regulation of BACE1 and Aβ42 peptide release is PPARγ-dependent. In conclusion, NPD1 bioactivity potently down regulates inflammatory signaling, amyloidogenic APP cleavage and apoptosis, underscoring the potential of this lipid mediator to rescue human brain cells in early stages of neurodegenerations.

## Introduction

Alzheimer's disease (AD) is a neurodegenerative disease characterized by progressive cognitive impairment and, at the cellular level, by synaptic damage, intracellular neurofibrillary tangles and beta-amyloid precursor protein (βAPP) processing dysfunction that leads to overabundance of the 42 amino acid amyloid-beta (Aβ42) peptide. Aβ42 promotes neuroinflammation, synaptic toxicity, and apoptosis, and it transitions extracellularly from an oligomer to an aggregate that, in turn, become a major component of senile plaques [1]–[6]. Aβ42 peptides are generated from βAPP via tandem cleavage by beta- and gamma- (β- and γ-) secretases; alternatively an alpha-secretase distintegrin and metalloproteinase 10 (ADAM10) cleaves βAPP to yield a soluble form of βAPP, sAPPα, via the non-amyloidogenic or neurotrophic pathway.

Docosahexaenoic acid (DHA; C22:6), an omega-3 essential fatty acid family member, is enriched in central nervous system (CNS), synaptic and other cellular membranes as an acyl chain of membrane phospholipids. DHA is involved in the building and function of the CNS, as well as synaptogenesis, cognition, neuroprotection, synaptic function and vision [7]–[10]. Current clinical trials favor a role for DHA in slowing cognitive decline in elderly individuals without dementia but not for the prevention or treatment of dementia, including AD [11], [12]. Deficiencies in DHA biosynthesis by the liver correlate with cognitive impairment in AD patients [13], supporting the significance of the liver supply of DHA to the CNS in neurodegenerative diseases [13], [14]. In AD transgenic mice dietary DHA restores cerebral blood volume, reduces Aβ deposition, and ameliorates Aβ pathology [15], [16].

The recent identification of the DHA-derived stereoselective mediator neuroprotectin D1 (NPD1; 10R,17S-dihydroxy-docosa-4Z,7Z,11E,15E,19Z hexaenoic acid) provides a specific mechanism to understand DHA-mediated modulation of neuroinflammation and neuroprotection. NPD1 elicits neuroprotective activity in brain ischemia-reperfusion and in oxidative-stressed retinal cells [17]–[19]. DNA microarray profiling suggests a down-regulation of pro-inflammatory genes as well as of some pro-apoptotic genes of the Bcl-2 gene family [9]. NPD1 further influences βAPP processing and decreases Aβ42 release [9], and its precursor DHA elicits an Aβ42-lowering effect both *in vitro* and *in vivo* [8], [20], [21]. In addition, free radical-mediated DHA peroxidation products accumulate during ischemia and neurodegeneration. These oxidation products in turn may form protein adducts and other cytotoxic molecules that promote further free radical injury [22]–[24].

The ligand-activated transcription factor peroxisome proliferator-activated receptor γ (PPARγ) regulates lipoprotein metabolism, adipogenesis and insulin sensitivity, and it has been implicated in AD [25]–[30]. PPARγ activation underlies some of DHA's anti-inflammatory actions [31]–[34]. Moreover, PPARγ is a potential NPD1 target since it has a fatty acid binding pocket for polyunsaturated fatty acids [31] and their derivatives, including DHA [35].

In the present study, we assessed DHA and NPD1 abundance in control and aged 3xTg-AD mouse hippocampus and used aging human neuronal-glial (HNG) primary cells to characterize NPD1 bioactivity on: neuroinflammatory events and apoptosis; to test the mechanism of NPD1-mediated regulation of Aβ42 secretion; and to assess the significance of PPARγ in the homeostatic bioactivity of NPD1. Here we provide evidence that, besides protecting against Aβ42-induced neurotoxicity via anti-inflammatory and anti-apoptotic bioactivity, NPD1 down-regulates the amyloidogenic processing of βAPP, thus reducing Aβ42 production. Moreover, NPD1 anti-amyloidogenic action through selective targeting of both the α- and β-secretase-mediated processing of βAPP and anti-amyloidogenic action are exerted through PPARγ receptor activation.

## Materials and Methods

Studies and procedures were performed according to National Institutes of Health and Canadian Council on Animal guidelines, and animal protocols were approved by the Institutional Animal Care and Use Committee at the Louisiana State University Health Sciences Center, New Orleans (IACUC #2705, IBC# 08126 and 082303), and by the Laval University Animal Ethics Committee (approval ID  = 07–113 and 07–061).

### Reagents and Antibodies

The following reagents and antibodies were obtained commercially and used without further purification: Aβ42 peptides (American Peptide, Sunnyvale, CA); antibodies for COX-2, APP-NT, APP-CT and β-actin (Sigma, St Louis, MS); antibodies for B94, ADAM9, ADAM10, BACE1 and PS1 (Santa Cruz Biotechnology, Santa Cruz, CA); antibodies for sAPPα and sAPPβ_sw_ (American Research Products, Belmont, MA); IRDyeTM 700 or 800 infrared dye-labeled secondary antibodies (Rockland Immunochemicals, Gilbertsville, PA); FITC fluorescein conjugated secondary antibody (BD Biosciences, San Jose, CA); Cy3 fluorescein conjugated secondary antibody (GE Healthcare, Piscataway, NJ). Additional BACE1 antibodies Ab2077 (Abcam, Cambridge MA); sc-73729 and sc-33711 (Santa Cruz); PA1-757 (Affinity Bioreagents, Rockford, IL) and 61-3E7 (MAB5308, Millipore, Billerica, MA) were used to analyze BACE1 abundance; the identity of BACE1 was also confirmed via C-terminal micro-sequencing (data not shown). DHA was obtained from (Cayman Chemical, Ann Arbor, MI) and stereochemically pure NPD1 was prepared via total organic synthesis and quantified according to reported chemical and physical procedures using molecular biology grade ethanol as vehicle (concentration 0.1 µg/µl ∼0.3 mM) [36], [37].

### 3xTg-AD Animals

**Figure 1:**
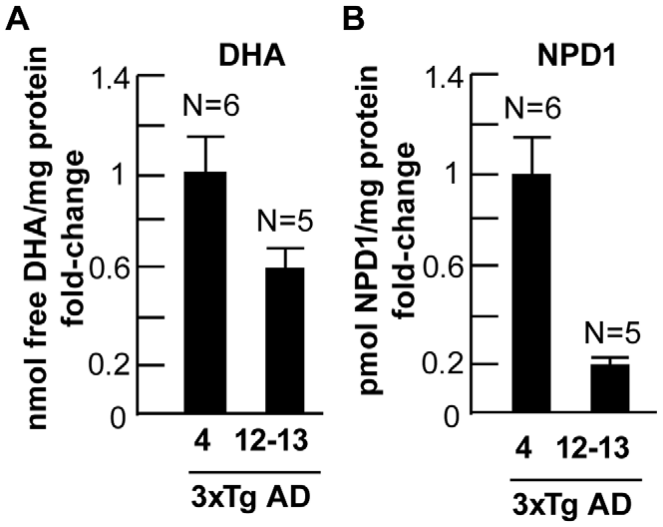
DHA and NPD1 analysis in 3xTg-AD hippocampus. DHA and NPD1 levels were analyzed using liquid chromatography-photodiode array-electrospray ionization-tandem mass spectrometry (LC-PDA-ESI-MS-MS) [9]. 3xTg-AD mouse brain hippocampus was sampled at 4 and 12–13 months and were compared to age-matched controls. DHA levels were reduced to 0.61-fold of control between 12–13 and 4 month old 3xTg AD mice (**A**); similarly NPD1 levels were reduced to 0.22-fold of control in 12–13 month old 3xTg-AD mice compared to 4 month 3xTg-AD mice (**B**). *p<0.05; ** *p*<0.01 (ANOVA).

3xTg-AD mice, harboring the PS1 (M146V), APP (Swe) and tau (P301L) human transgenes were reared according to established protocols and received control diets (Teklad 2018, rodent diet; Harlan Teklad, Inianapolis IN) [38], [39]. Non-transgenic mice used here were littermates from the original PS1-knockin mice and are on the same background as 3xTg-AD mice (C57BL6/129SvJ) [39]. Animals (N = 5 to 6 per group) were sacrificed at 4 and 12–13 months and the frontal lobe, rostro-temporal lobe and hippocampus were isolated and analyzed for DHA and NPD1 using LC-PDA-ESI-MS-MS as previously reported [9], [17], [18], [37] (**Figure 1**).

### Human neuronal-glial (HNG) Cell Culture

We used stressed primary human neuronal-glial co-culture (HNG) as a cellular model to address NPD1-mediated signaling and mechanistic questions relevant to AD [9], [27], [40], [41]. In brief, human neural progenitor cells (Lonza, Allendale, NJ) were initially plated as free-floating aggregates (neurospheres) in a 75 cm^2^ uncoated plastic flask in neural progenitor basal medium (NPBM) supplemented with human recombinant basic fibroblast growth factor (hbFGF), human recombinant epidermal growth factor (hEGF) and human leukemia inhibitory factor (hLIF). Differentiation into HNG cells was induced by plating neurospheres onto 6-well plates pre-coated with poly-L-ornithine and cultured in neurobasal medium supplemented with 1×B27 (Invitrogen, Carlsbad, CA) and 1% of FBS. Cells were subsequently cultured for 1 week until 80% confluence at 37°C in a humidified 5% CO_2_ atmosphere incubator; 24 hours after plating HNG cells then strongly adhere to the plate bottom [9], [27].

### Plasmid Constructs and Transient Transfection of HNG Cells

Plasmid containing APP695 cDNA bearing the Swedish mutation APP_sw_ (Swedish double mutation APP695_sw_, K595N, M596L) was a generous gift from Dr. T Golde of the Mayo Clinic (Jacksonville, FL). cDNA clones of full length hBACE1 genes were from Open Biosystem (Huntsville, AL). HNG cells were plated in 6-well plates at 80% confluence and transiently transfected using Fugene HD transfection reagent (Roche Applied Science, Indianapolis, IN) with 2 µg per well of hAPP695_sw_ plasmid DNA alone or together with pEGFP (green fluorescent protein; BD Biosciences-Clontech), hPPARγ, or hBACE1 at a DNA (µg):reagent (µl) ratio of 1∶3. After 24 h, cells were typically incubated with 0, 50, 100 or 500 nM NPD1 or vehicle for 48 h before assay.

### Small Interfering RNA-mediated Gene Silencing

HNG cells were transfected with predesigned siRNA (Santa Cruz Biotechnology) to knock down human ADAM9 or ADAM10 mRNA. HNG cells over-expressing βAPP_sw_ were transfected with a total of 60 pmol of ADAM9, ADAM10 or control siRNA using Lipofectamine 2000 transfection reagent (Invitrogen) and cultured for 24 h. The medium was replaced with a fresh one containing 500 nM of NPD1 and cells were cultured for another 48 h before assay.

### Aβ42 Oligomer Preparation

Aβ42 peptides were initially solubilized in hexafluoroisopropanol (HFIP) (Sigma), aliquoted, and stored at −20°C as an HFIP film [42]. After evaporating HFIP, aliquoted peptide was re-suspended with DMSO to 5 mM and diluted with phenol red free F12 media (Invitrogen) to a concentration of 100 µM. Peptide for the oligomer preparation was incubated at 4°C for 24 h prior to use [42]. The oligomeric status of Aβ was verified by sodium dodecyl sulfate polyacrylamide gel electrophoresis (SDS-PAGE; Figure S1).

### Immunocytochemistry and Imaging Analysis

HNG cells cultured on 8-well chamber slides (BD Biosciences, San Jose, CA) were fixed with 4% paraformaldehyde, then permeabilized and blocked with 0.125% Triton X-100 (Sigma) and 2% normal goat serum (GE Healthcare) in PBS at room temperature (RT) for 1 h. Cells were incubated overnight at 4°C with antibodies for β-tubulin III, GFAP, COX-2, B94 or APP-NT. Cells were washed 3 times with PBS and incubated for 3 h at RT with secondary antibodies conjugated with Cy3 or FITC fluorescein. After washing and drying, slides were applied with mounting medium (Vector Laboratories, Burlingame, CA) and observed under Zeiss Axioplan Inverted Deconvolution Fluorescent Microscope (Carl Zeiss, Oberkochen, Germany). Positively-stained cells were quantified using the manual counter function of the NIH ImageJ software.

### Mediator lipidomic analysis

Lipids were extracted by homogenization of cells or tissues in chloroform/methanol and stored under nitrogen at −80°C [17], [18], [36]. For quantification, lipid extracts were supplemented with deuterated labeled internal standards, purified by solid-phase extraction, and loaded onto a Biobasic-AX column (Thermo-Hypersil-Keystone; 100 mm ×2.1 mm; 5-µm particle sizes) run with a 45-min gradient protocol, starting with solvent solution A (40∶60∶0∶01 methanol:water:acetic acid, pH 4.5; 300 µl/min); the gradient typically reached 100% solvent B (99.99∶0.01 methanol:acetic acid) in 30 min, and was then run isocratically for 5 min. A TSQ Quantum (Thermo-Finnigan) triple quadrupole mass spectrometer and electrospray ionization was used with spray voltage of 3 kV and N_2_ sheath gas (35 cm^3^/min, 350°C). Parent ions were detected on full-scan mode on the Q1 quadrupole. Quantitative analysis was performed by selective reaction monitoring. The Q2 collision gas was argon at 1.5 mTorr, and daughter ions were detected on Q3. Selected parent/daughter ion pairs for NPD1 and unesterified DHA were typically 359/153 *m/z* and 327/283 *m/z*, respectively. Calibration curves for NPD1 and DHA (Cayman Chemical) were acquired; NPD1 was generated via biogenic synthesis using soybean lipoxygenase and DHA, purified by HPLC, and characterized by LC-PDA-ESI-MS-MS according to reported biophysical criteria [9], [17].

### MTT cell viability assay, Hoechst staining, TUNEL assay and caspase-3 activity assay

Cell viability was measured by 3-[4,5-dimethylthiazol-2-yl]-2,5-diphenyl tetrazolium bromide (MTT) reduction assay (Sigma). HNG cells were incubated with 5 µM of Aβ42 in the absence or presence of 50 nM of NPD1 for 48 h. MTT was added to a final concentration of 0.5 mg/ml and incubated for 2 h. Medium was then removed and equal volumes of isopropanol were added to dissolve the resulting formazan crystals. Absorbance was spectrophotometrically measured with a SpectraMax Microplate Reader (Molecular Devices, Sunnyvale, CA) at 570 nm. HNG cells were further incubated with 2 µM Hoechst 33258 (Invitrogen) for 45 min at 37°C before imaging. Cells were then viewed by using a Nikon DIAPHOT 200 microscope under UV fluorescence. Images were recorded by a Hamamatsu Color Chilled 3CCD camera and PHOTOSHOP 7.0 software. Positively stained cells were counted manually using ImageJ software. The apoptotic nuclei containing free 3′-OH termini were detected using DeadEnd Fluorometric TUNEL Kit (Promega, Madison, WI). Samples were analyzed under a Zeiss Deconvolution Microscope. Caspase-3 activity from cell lysates was detected using Caspase 3 Colorimetric Assay Kit (Sigma). The absorbance was measured at 405 nm using a SpectraMax Microplate Reader.

### Total RNA Extraction and RT-PCR

HNG cells were lysed and total RNA was extracted with TRIzol (Invitrogen). RNA quality and quantity were analyzed by using a 2100 Bioanalyzer (Agilent Technologies, Santa Clara, CA). 28S/18S ratio for each RNA sample was typically greater than 1.8. For reverse transcription, a Superscript III First-Strand SuperMix (Invitrogen) was used. 1 µg of total RNA was used as a template to react with 10 µl of 2×RT Reaction Mix and 2 µl RT Enzyme Mix. Final total volume was 20 µl. Samples were incubated at 25°C for 10 min and then 50°C for 30 min. Reactions were stopped by heating to 85°C for 5 min, and RT product was amplified with Phusion High Fidelity DNA Polymerase in a GeneAmp PCR System 9700 (Applied Biosystems, Foster City, CA). The primers used in these experiments (Integrated DNA Technologies, Coralville, IA) and their sequences are as follows: 5′-TTTGATGATGGCGTACTTGG-3′, 5′-AGTTTGTCCCCAGATGTTGC-3′ for ADAM10; 5′-TACAATGCTGACTATGGCTAC-3′, 5′-CTGATGCGTGAAGTGCTG-3′ for COX-2; 5′-CAAAGTAGACCTGCCCAGAC-3′, 5′-GACCTCTCTCTAATCAGCCC-3′ for TNF-α; 5′-TGAGGACCTGAAG CCACTGTTCAA-3′, 5′-TGCGCTTGACCTCACTGTTGGATA-3′ for B94 and 5′-AGATGTGGATCA GCAAGCAGGAGT-3′, 5′-GCAATCAAAGTCCTCGGCCACATT-3′ for β-actin (internal control; Lukiw and Pogue 2007; Lukiw et al., 2005). The PCR consisted of initial incubation at 98°C for 45 s, denaturation at 98°C for 10 s, annealing at 56°C for 30 s and extension at 72°C for 20 s, for 35 cycles, and final extension at 72°C for 7 min. PCR products were further analyzed on 1.5% agarose gels; relative band intensity was quantified using Quality One software (Invitrogen).

### SDS-PAGE and Western Blotting

Conditioned media were collected from cultured HN cells after various treatments and protease inhibitor cocktail (Sigma) was added to 1% final concentration [9], [27]. Cells were then washed twice with ice-cold DPBS and lysed and harvested in RIPA buffer (Sigma) supplemented with 2% protease inhibitor cocktail, then centrifuged at 10,000× g for 15 min at 4°C. Supernatants were collected and quantified using Bio-Rad's (Hercules, CA) DC Protein Assay kit. 30 µg of cell lysate or 20 µl - of conditioned media were electrophoresed on 4–15% Tris-HCl gradient gels at 100 V for 80 min or 10–20% Tris-Tricine gels for the detection of CTFs at 50 mA for 120 min. Proteins were transferred to an Immobilon FL PVDF membrane (Millipore, Billerica, MA) at 100 V for 60 min. Membranes were incubated with primary antibody overnight at 4°C, followed by incubation with IRDye 800 or Alexa 680-conjugated secondary antibodies for 5 h at RT. After repeated washing with Tris-buffered saline, the membrane was then visualized by the Odyssey Infrared Imaging System (LI-COR, Lincoln, NE).

### Sandwich ELISA Analysis of TNFα and Aβ42

Secreted TNF-α, Aβ42 and total Aβ were detected using a Human TNF ELISA Kit (BD Biosciences, San Jose, CA), a human amyloid β 42 ELISA kit (Sigma) and a human amyloid β (1-x) assay kit (American Research Products), respectively. After reactions, the plates were immediately measured at 450 nm by a SpectraMax Microplate Reader.

### Human Preadipocyte Differentiation Assay

Human preadipocytes maintenance and differentiation procedures were performed according to the manufacturer's instructions with modifications (Zen-Bio, Research Triangle Park, NC). Briefly, upon the initiation of the differentiation assay, preadipocytes were incubated in adipocyte medium supplemented with IBMX (0.5 mM) and NPD1, DHA or vehicle. A concentration range of 0.1–5 µM of each lipid was used. After 3-day incubation, the cell medium was replaced with the adipocyte medium without IBMX. Eight days after vehicle or lipid treatment, the media was removed and the cells were fixed with formalin (7% formaldehyde in PBS). Cells were then stained with Oil Red O (Sigma, Saint Louis, MS) and pictures were taken with a Nikon Eclipse TS100 inverted microscope (Nikon USA, Melville, NY). The Oil Red O-stained total lipid was then eluted with 100% isopropanol and quantified by measuring OD value at 500 nm with a SpectraMax Microplate Reader.

### Cell-based PPARγ Transactivation Assay

The two plasmids used for the transactivation assay (PPARγ-GAL4 and MH100-tk-luc) were kindly provided by Dr. Ronald Evans of Salk Institute (La Jolla, CA) [32]. Luciferase assay was performed using Promega's Luciferase Assay System. Light units from firefly luciferase and β-galactosidase activities were measured in a Luminoskan Ascent microplate luminometer (Thermo Fisher Scientific, Waltham, MA). Luciferase values were expressed as relative light units and normalized to the level of β-galactosidase activity. Changes in PPARγ activity were expressed as “fold induction” relative to the vehicle control values (Figure S2).

### Statistical Analysis

All experiments were repeated at least three times using independent culture preparations. Data are presented as mean ± S.E. Quantitative data were statistically analyzed by one-way analysis of variance (ANOVA) followed by pair-wise comparisons using the Fisher's least significant difference test. A *p*<0.05 was considered significant.

## Results

### DHA and NPD1 deficits in 3xTg-AD mouse hippocampus

DHA and NPD1 levels were assayed in the hippocampus of 3xTg-AD mice, harboring the PS1 (M146V), APP (Swe) and tau (P301L) human transgenes that model several human AD features [38], [39]. DHA and NPD1 levels were analyzed using LC-PDA-ESI-MS-MS-based lipidomic analysis as previously described (**Figure 1**) [9], [18]. Both DHA and NPD1 showed age-related changes in 4-month old versus 12–13 month old 3xTg-AD animals. DHA concentration in the hippocampus was found to be reduced 2-fold between 4-month old control versus 4-month 3xTg-AD animals, and 3-fold between 4 and 12-13 month old control animals (**Figure 1A**). NPD1 in the hippocampus showed dramatic reductions both in aging control animals; a 12-fold reduction between 4 and 12–13 month controls and a 3-fold reduction between 4 and 12–13 month 3xTg-AD mice (**Figure 1B** **).**

### NPD1 protects HNG cells from Aβ42-induced apoptosis

**Figure 2:**
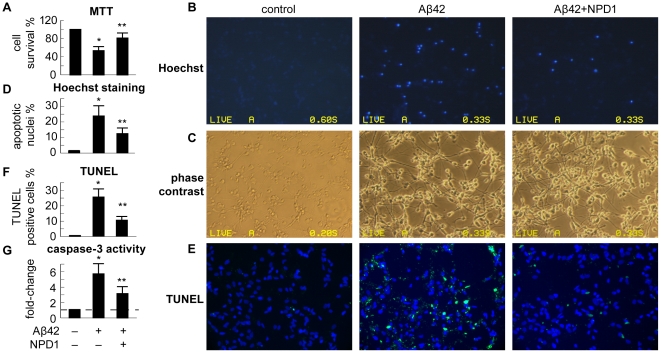
NPD1 promotes cell survival in Aβ42 oligomer stressed-human HNG cells in primary culture. HNG cells were treated for 48 h with 5 µM of Aβ42 oligomer in the absence or presence of NPD1 (50 nM). (**A**) NPD1 promotes HNG cell survival in response to Aβ42 neurotoxicity as shown by MTT cell viability assay following reduction of the tetrazolium salt MTT (Sigma-Aldrich). Results, expressed as the percentage of cell survival, are the means ± SEM of three experiments performed in triplicate. Untreated cells were 100% viable. Panels (**B-G**) depict anti-apoptotic effect of NPD1 by combining Hoechst 33258 staining, phase contrast microscopy, TUNEL assay and caspase-3 activity assay. (**B**) Upper panel illustrates the appearance of Hoechst 33258 positive cells upon different treatments (20× magnification); (**C**) middle panel shows phase-contrast images of corresponding HNG cells. (**D**) Number of Hoechst 33258-positive apoptotic nuclei per 100 of total cells (n = 5). (**E**) TUNEL (green fluorescence) assay and (DAPI) (blue) staining (20× magnification) of HNG cells. (**F**) Percentage of TUNEL-positive cellular nuclei in each treatment group (n = 5). (**G**) Caspase-3 activity of HNG cells after Aβ42 peptide and NPD1 treatment; dashed horizontal line at 1.0 indicates control caspase-3 activity for ease of comparison; (n = 3). Data are expressed as means ± SEM. *p<0.01 vs. control; **p<0.01 vs. Aβ42 peptide.

**Figure 3:**
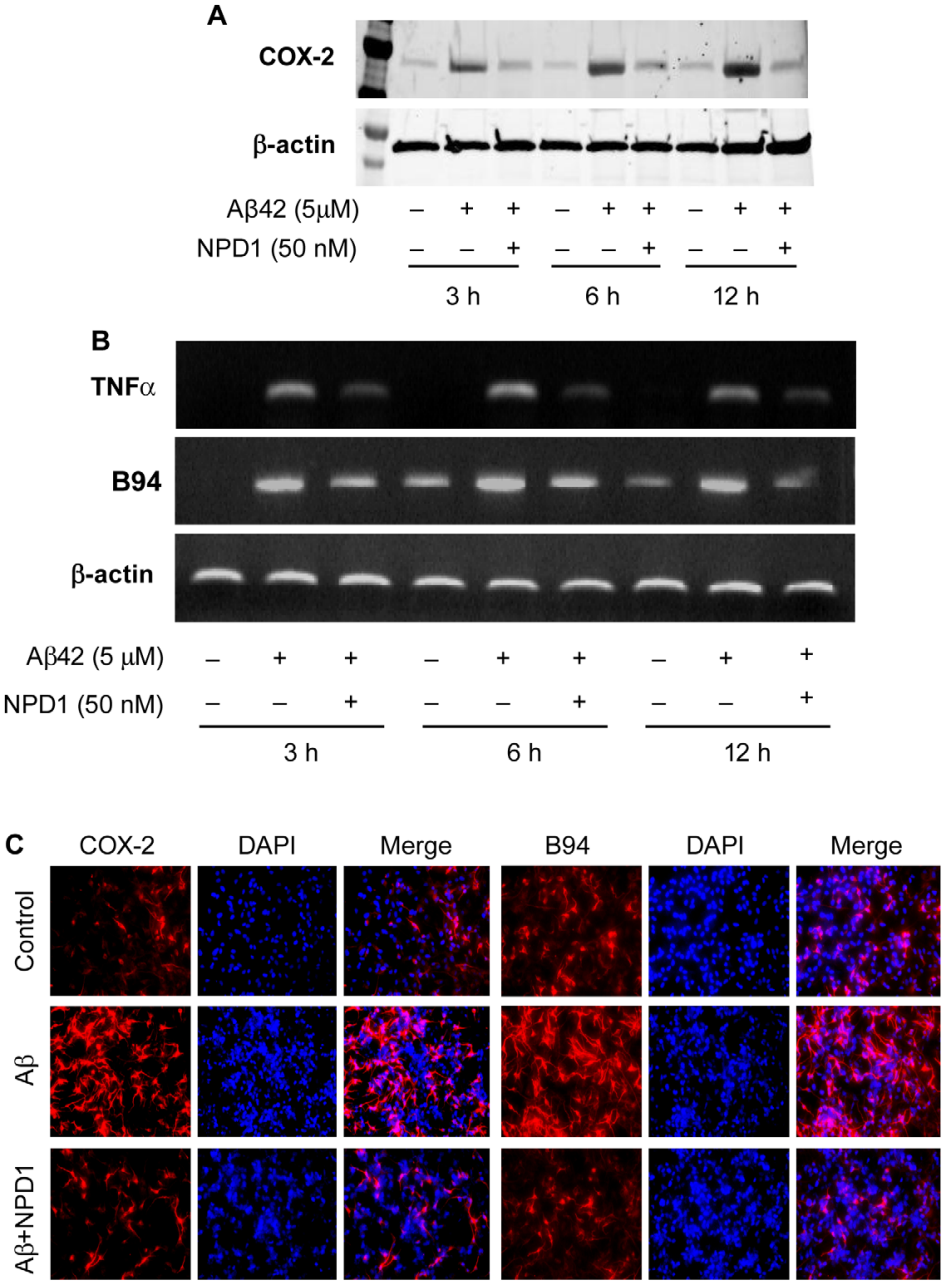
NPD1 down-regulates the expression of the pro-inflammatory genes COX-2, TNFα and B94, compared to β-actin levels in the same sample, in response to Aβ42 oligomer (5 µM); rescue by NPD1 (50 nM). (A) COX-2 and β-actin mRNA expression was detected by RT-PCR followed by agarose gel electrophoresis. (B) Western blot detection of TNFα, B94 and β-actin expression in control or Aβ42 peptide-stressed HNG cells at 3, 6 and 12 h in the presence or absence of NPD1. (C) Immuno-fluorescent staining of COX-2 or B94 (red). Nuclei were visualized using DAPI (blue) (20× magnification). Quantitative analysis of COX-2 and B94 gene expression (n = 3 to 5; see **Figure 5**).

Phase contrast and immunofluorescence of differentiated HNG cells expressing the neuronal marker β-tubulin III and the astrocyte marker glial fibrillary acidic protein (GFAP) revealed neuronal-glial co-cultures containing about 50% neurons under these conditions (**Figure 2**). NPD1 was shown to counteract Aβ42 oligomer-induced apoptosis in HNG cells using MTT, Hoechst 33258 staining, TUNEL and assay of caspase-3 activity (**Figure 3A–G**). These assays showed that over 48 h Aβ42 oligomer triggers about 50% cell death with concomitant nuclear compaction and striking apoptotic changes (**Figure 3**). Aβ42 peptides also enhanced caspase-3 activity at least 6-fold, an effect that was reduced in the presence of NPD1. Co-incubation of 50 nM NPD1 with Aβ42 oligomer resulted in enhanced cell viability and attenuation of Aβ42 peptide-mediated apoptosis and cytotoxicity (**Figure 3**).

### NPD1 down-regulates Aβ42 oligomer-induced pro-inflammatory gene expression

**Figure 4:**
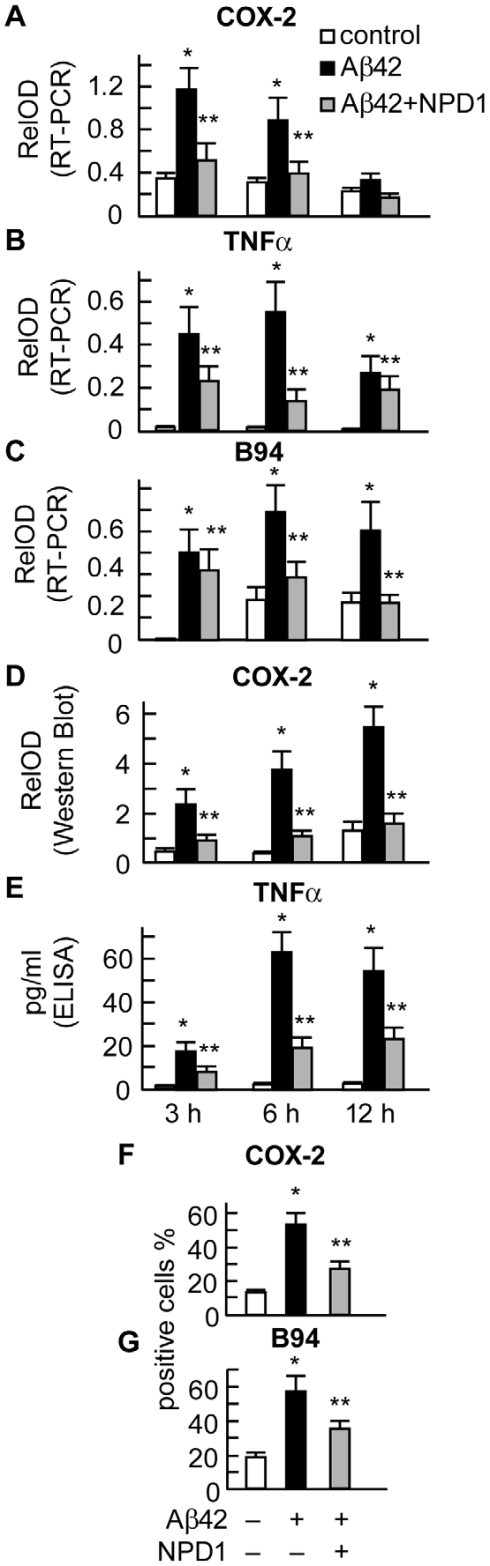
NPD1 down-regulates Aβ42-induced expression of pro-inflammatory genes COX-2, TNFα and B94 at both the mRNA (RT-PCR) and protein (Western blot or ELISA) level. HNG cells were incubated with 5 µM of Aβ42 oligomer in the absence or presence of NPD1 (50 nM) for 3, 6 and 12 h. NPD1 suppressed Aβ42 peptide-induced mRNA expression of COX-2, TNFα and B94. (**A–C**) mRNA expression was detected by RT-PCR followed by agarose gel electrophoresis (**see Figure 4**); (D,E) NPD1 reduced COX-2 and TNFα protein abundance in response to Aβ42 stress; (**E**) Time course of TNFα secretion as detected by ELISA (n = 3); (**F,G**) NPD1 reduced the number of COX-2 and B94 positive cells after Aβ42 peptide-induced stress. In **F** and **G** HNG cells were incubated for 24 h under the indicated treatments. Results are means ± SEM. *p<0.01 vs. control; **p<0.01 vs. Aβ42 peptide-treated.

**Figure 5:**
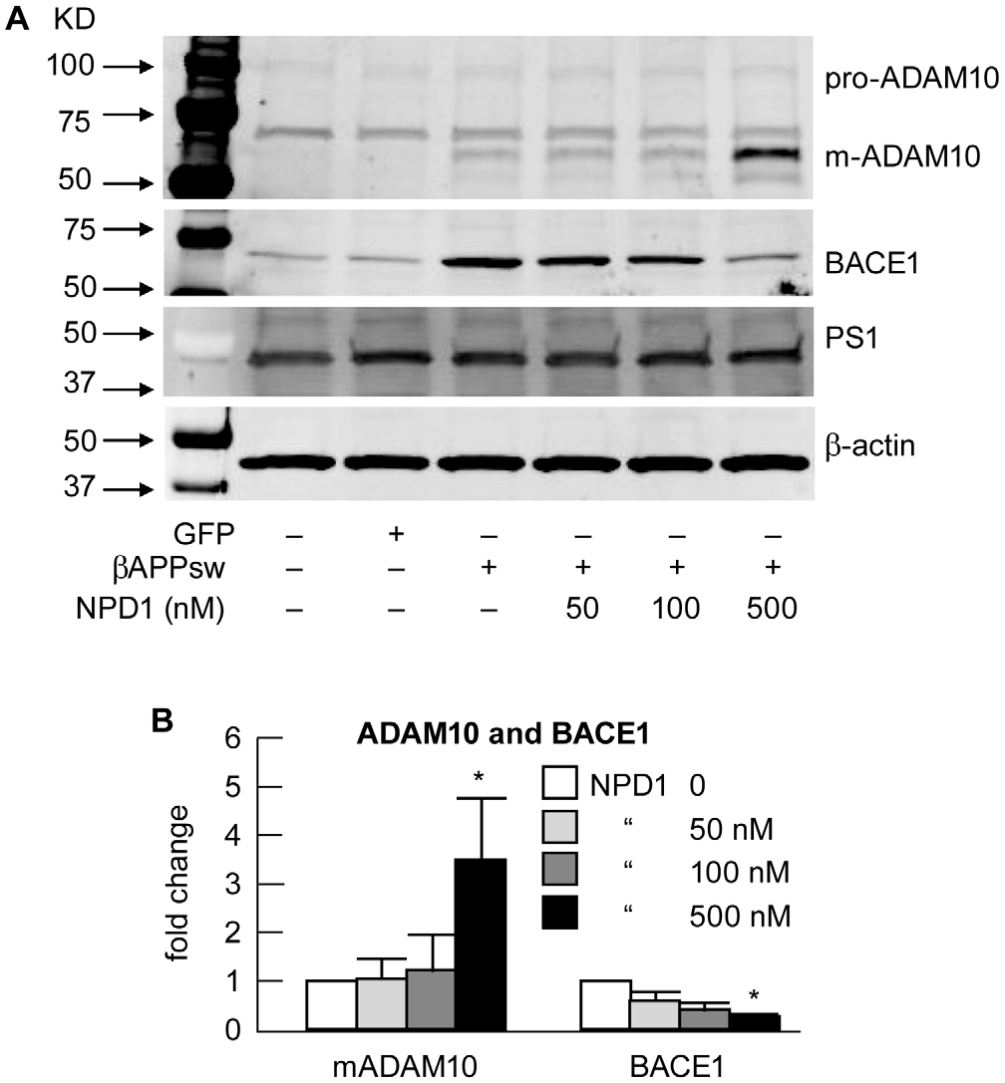
HNG cells transfected with βAPP_sw_ and treated with NPD1 - effects on precursor α-secretase (pro-ADAM10), mature ADAM10 (m-ADAM10), β-secretase (BACE1) and γ-secretase (PS1). (**A**) Control or HNG cells over-expressing βAPP_sw_ were incubated with increasing doses (0, 50, 100 and 500 nM, respectively) of NPD1 before cell lysates were harvested and subjected to Western blotting for the precursor of ADAM10 (pro-ADAM10), mature ADAM10 (m-ADAM10), BACE1 and PS1 using the levels of β-actin as a control in the same sample; (**B**) Quantitation of gel bands in (A); NPD1 activates m-ADAM10 while inhibiting BACE1 expression; quantification of m-ADAM10 and BACE1 expression by Western blotting analysis after normalization to β-actin; results are means ± SEM (n = 3); *p<0.01 vs. βAPP_sw_ control.

Our previous DNA microarray-based analysis suggested anti-inflammatory bioactivity of NPD1 in HNG cells, as shown by their attenuation of Aβ42 peptide-induced elevation of the pro-inflammatory genes COX-2, TNF-α and B94 [9]. Here we extended these studies by exploring NPD1 actions at both the mRNA and protein levels using RT-PCR, Western assay, ELISA assay and immunocytochemistry (**Figures 3** **and** **4**). The relative basal abundance of TNF-α mRNA was low, B94 mRNA increased during incubation at 6 h, and constitutive expression of COX-2 mRNA occurred during incubation. Aβ42 increased mRNA abundance of COX-2, TNF-α and B94 at 3, 6 and 12 h (**Figure 4A,B**). COX-2 mRNA stood out because it displayed immediate early-inducible gene behavior upon Aβ42 peptide exposure [9]. Protein expression of cellular COX-2, TNF-α secreted to the incubation media, and immunocytochemistry of COX-2 and B94 showed Aβ42-stimulated enhancement and NPD1 (50 nM) markedly reduced Aβ42 oligomer-stimulated mRNA increases as well as COX-2, TNF-α and B94 protein expression (**Figure 4C**). NPD1 therefore elicits potent down-regulation in the expression of a specific set of pro-inflammatory and pro-apoptotic genes known to be up-regulated in AD hippocampus and in stressed HNG cell models of AD [9], [41], [43]–[46]. Messenger RNA, Western, ELISA and immunohistochemisty data are presented in **Figures 4** and **5**.

### NPD1 represses amyloidogenic processing of βAPP with concomitant stimulation of non-amyloidogenic processing

**Figure 6:**
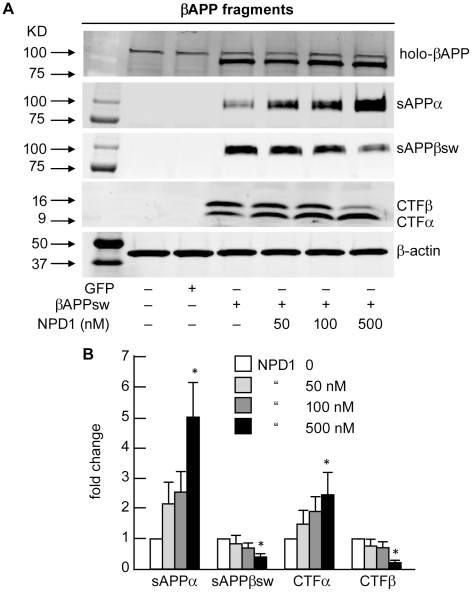
NPD1 shifts βAPP processing from the amyloidogenic to the non-amyloidogenic pathway. (**A**) Control or HNG cells over-expressing βAPP_sw_ were treated with increasing concentrations (0, 50, 100, 500 nM) of NPD1 for 48 h and subjected to Western blot detection of holo-βAPP (βAPP holoenzyme), sAPPα, sAPPβ_sw_, CTFα and CTFβ in comparison to β-actin levels in the same sample; (**B**) Quantification of gel bands in (**A**) analyzing βAPP fragments with increasing doses of NPD1. Results are means ± SEM (n = 4); *p<0.01 vs. βAPP_sw_ control.

**Figure 7:**
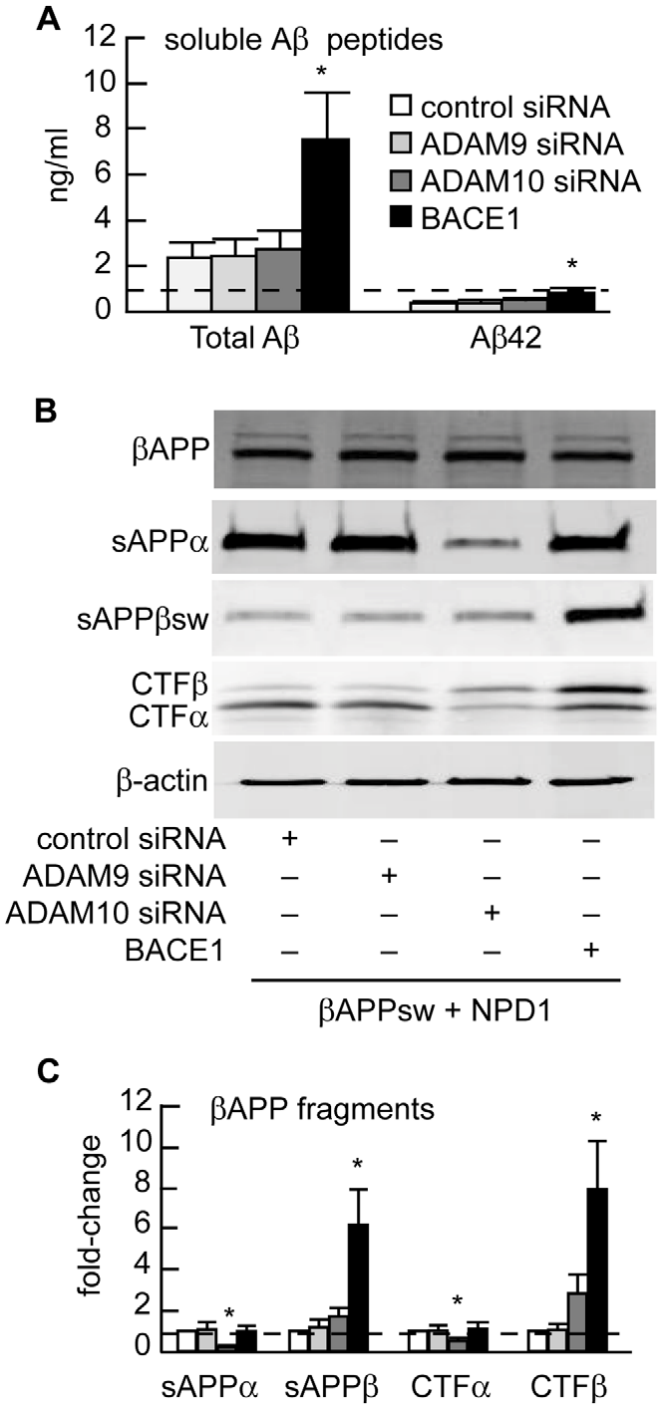
BACE1 and ADAM10 are required in NPD1-regulated βAPP processing into the release of soluble Aβ peptides and Aβ42 peptides (ng/ml cell culture medium). (**A**) Effects of control siRNA, ADAM9 siRNA or ADAM10 siRNA on shedding of total Aβ peptides or Aβ42 peptides into the HNG cell growth medium. (**B**) HNG cells over-expressing βAPP_sw_ were co-transfected with control siRNA or siRNA specifically targeting ADAM9 or ADAM10, or with BACE1 plasmid DNA for 48 h in the presence of 500 nM of NPD1. (**C**) Quantification of gel bands in (**B**) analyzing βAPP fragments with increasing doses of NPD1. Results are means ± SEM (N = 4); *p<0.01 vs. βAPP_sw_ control. Effects of different treatments are measured by ELISA for total Aβ peptides or Aβ42 peptides (n = 5), or by Western blotting (n = 3); * *p*<0.01 vs. βAPP_sw_ control.

Aβ42-peptides are secreted from human brain cells as they age or in response to physiological stress [4], [9], [27], [47], [48]. The processing of βAPP holoenzyme and secretion of βAPP fragments is controlled in large part by alpha-, beta- and gamma- (α-,β- and γ-) secretases [3], [4]. To assess the effects of NPD1 on secretase-mediated Aβ42 peptide generation, we used HNG cells transiently-transfected with βAPP_sw_ and assayed for the abundance of the α-secretase–generating enzymes precursor-ADAM10 (pro-ADAM10), mature-ADAM10 (m-ADAM10), β-amyloid cleavage enzyme (BACE1) and the gamma-secretase presenilin-1 (PS1) (**Figure 6**). Western blot analysis revealed that the steady-level of BACE1 was reduced by 500 nM of NPD1. Meanwhile, the active and mature form of ADAM10 (m-ADAM10), the putative α-secretase, was dose-dependently increased in response to NPD1. We did not find changes in the pro-ADAM10, the inactive precursor or in the mRNA abundance of ADAM10 (data not shown; **Figure 6**). The undergoing changes in these two secretases are in agreement with alterations in Aβ42 peptide abundance, and in other cleavage products of βAPP (**Figure 7**). Interestingly, both m-ADAM10 and BACE1 levels were elevated in βAPP-over-expressing cells (**Figure 6**). Presenilin 1 (PS1), the main catalytic component for γ-secretase, remains unchanged after different βAPP_sw_ or NPD1 treatments (**Figure 6**). This same pattern was also seen in their C-terminal counterparts, CTFβ and CTFα; importantly, no change was observed in the steady-state level of the neural cell abundant βAPP (holo-βAPP; see **Figure 7A**). NPD1-mediated up-regulation of m-ADAM-10 and down-regulation of BACE1 was apparent with maximal effect at 500 nM, the highest concentration used in these experiments (**Figures 6** **and** **7**).

### Quantification of βAPP Fragments

As Aβ42 peptide generation is regulated by differential βAPP processing, NPD1-mediated Aβ42 peptide reduction is due to altered βAPP processing, and thereby altered βAPP cleavage products should confirm these catabolic outcomes. To test this idea, we used HNG cells over-expressing βAPP_sw_, and measured levels of N-terminal (sAPPα and sAPPβ_sw_) and C-terminal fragments (CTFα and CTFβ) of βAPP as well as holo-βAPP protein upon exposure to increasing concentrations of NPD1. We show that NPD1 lowers sAPPβ_sw_ secretion and elevates sAPPα in a dose-dependent manner (**Figure 7**). This observation is paralleled by a decrease in CTFβ and an increase in CTFα in the same cellular fractions and a significant 3.4-fold increase in mADAM10 (**Figure 7**).

### Silencing of ADAM9 and ADAM10 and overexpression of BACE1

**Figure 8:**
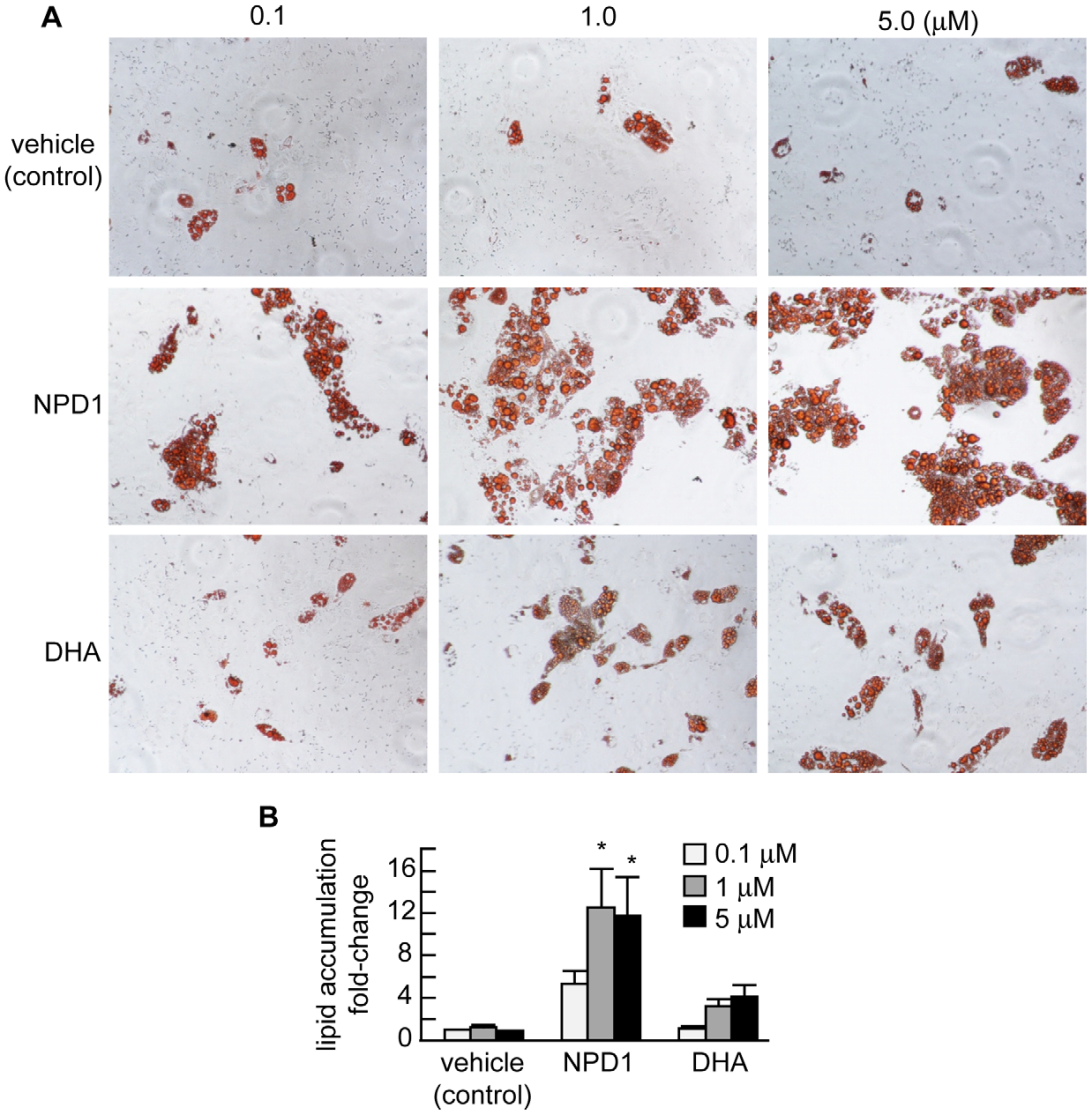
Human pre-adipocytes were cultured according to the manufacturer's instructions (Zen-Bio, Research Triangle Park, NC) (A). Upon initiation of the differentiation assay, pre-adipocytes were incubated in adipocyte medium supplemented with vehicle (control), NPD1 or DHA (see Materials and Methods). Results quantified in bar graph format indicate significant up-regulation of lipid accumulation in NPD1-treated, in contrast to DHA-treated, human pre-adipocyte cells (**B**).

Collectively, these data suggests the participation and modulation of BACE1 and ADAM10 activities in NPD1-mediated regulation of βAPP processing. Just like ADAM10, ADAM9 is also endowed with α-secretase activity [6], [49], and changes in BACE1 abundance may also contribute to Aβ42 peptide reduction. We therefore investigated whether ADAM 9, ADAM10 and BACE1 are essential to NPD1's regulation of βAPP processing by knocking down siRNA-targeted ADAM9 and ADAM10 genes. We also over-expressed BACE1 by transfecting HNG cells with a plasmid bearing the human BACE1 full length cDNA. We then measured total βAPP and other βAPP cleavage fragments in the presence of NPD1 with or without ADAM 9 siRNA or ADAM10 siRNA knockdown or BACE1 over-expression. As seen in **Figure 8**, when compared to controls (control siRNA) no changes occurred in ADAM9 siRNA group while ADAM10 siRNA-mediated knockdown almost completely abrogated the induction of sAPPα and CTFα by NPD1. Similarly, moderate over-expression of BACE1 overturned NPD1-induced reduction in Aβ42 peptides along with sAPPβ_sw_ and CTFβ fragments. These results in combination with those shown in **Figures 6** **and** **7** strongly suggests that NPD1's regulatory action targeting βAPP processing may be mediated in part through coordinated up-regulation of the α-secretase ADAM10 and down-regulation of BACE1 enzymatic activity.

### NPD1 is a PPARγ activator

**Figure 9:**
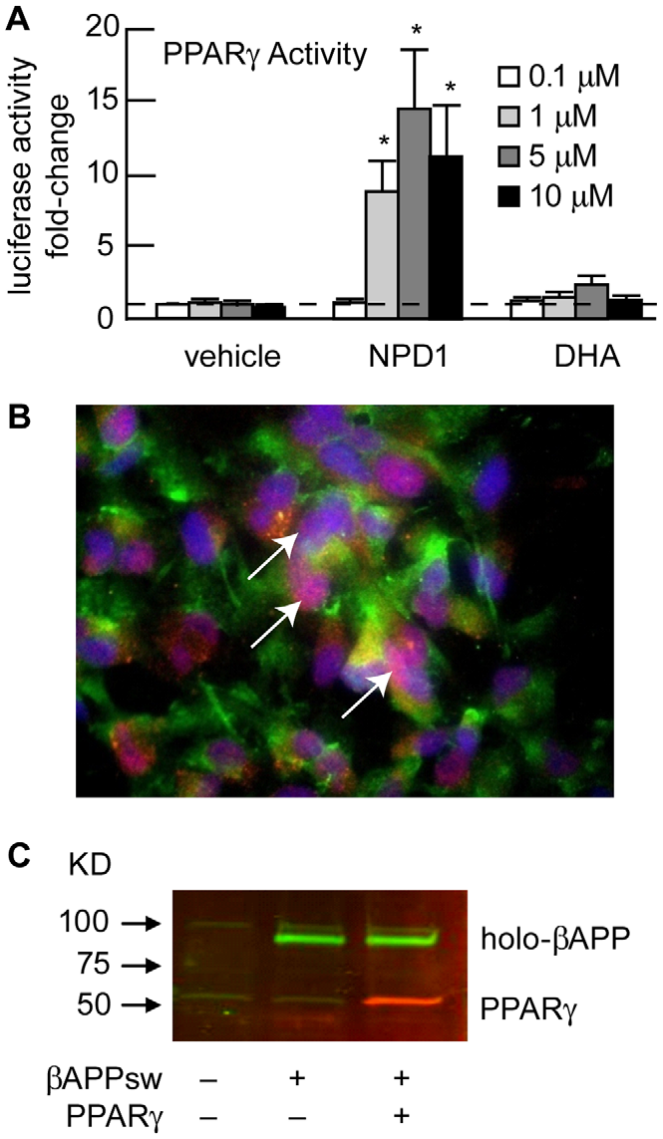
NPD1 activates PPARγ. (**A**) PPARγ activities upon incubation with NPD1 or DHA are shown in bar graph format. The activation of PPARγ by NPD1 and DHA was assessed using a cell-based luciferase reporter transactivation assay after incubation with increasing concentrations (0.1, 1.0, 5.0 and 10.0 µM) of NPD1 or DHA for 24 h. Luciferase activity was normalized to β-gal activity (transfection efficiency control) and results were expressed as fold-change of induction relative to vehicle treated HNG cells (n = 4). Horizontal dashed line at 1.0 indicates control PPARγ levels at 1 µM for ease of comparison; *p<0.01 vs. corresponding vehicle controls. (**B**) Co-transfection of βAPP_sw_ and PPARγ in HNG cells; immunofluorescence detection of βAPP_sw_ (green; λ = 530 nm) and PPARγ (red; λ = 670 nm) in HNG cells over-expressing both proteins; HNG nuclei are stained with DAPI (blue; λ = 470 nm); overlap of PPARγ and HNG cell nuclear signal (violet; λ = 420 nm; arrows) indicates nuclear association of PPARγ; 40x magnification. (**C**) Two-color Western blot with Odyssey infrared imaging showing over-expression of both βAPP_sw_ (green) and PPARγ (red) in whole HNG cell extracts.

**Figure 10:**
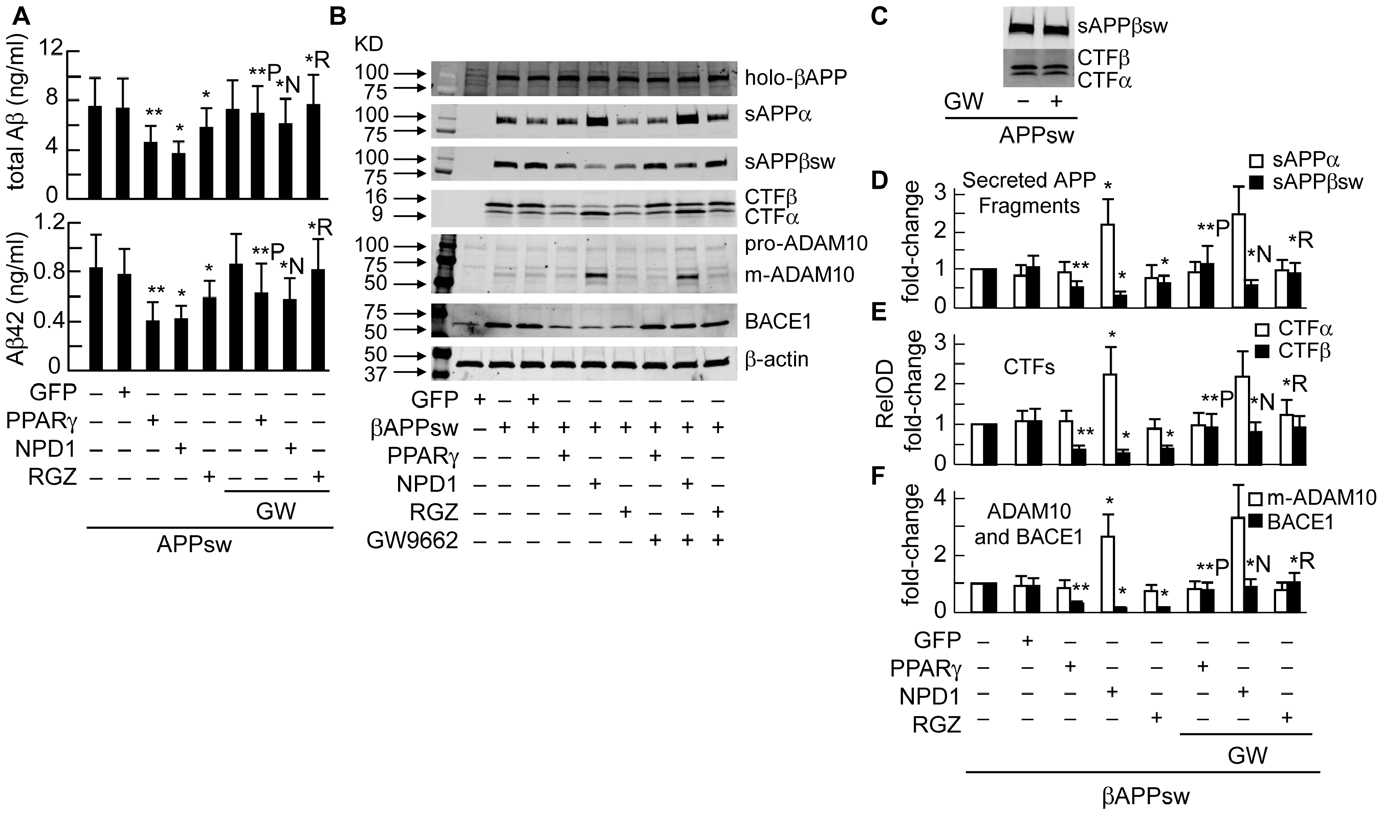
PPARγ activation is required for anti-amyloidogenic effect of NPD1 but not for activation of ADAM10. HNG cells over-expressing βAPP_sw_ were co-transfected with 2.0 µg of pEGFP or PPARγ cDNA or incubated with 0.5 µM of NPD1 or rosiglitazone (RGZ) in the absence or presence of 2 µM of GW9662 for 48 h before being harvested for assay. (**A**) ELISA assay of total Aβ and Aβ42 peptides (ng/ml) from conditioned media of HNG cells with different treatments; (**B**) Western blot analysis of βAPP fragments as well as ADAM10 and BACE1 from HNG cells under different conditions as indicated in the lower part of panel (**B**) in these Western images; (**C**) Western analysis of sAPPβ_sw_ or CTFs from HNG cells over-expressing βAPP_sw_ in the absence or presence of 2 µM of GW9662; (**D–F**) Quantification of βAPP fragments, ADAM10 and BACE1 levels based on Western results (n = 3). *p<0.01 vs. βAPP_sw_ control; **p<0.01 vs βAPP_sw_ + pEGFP co-transfection; *N *p*<0.01 vs. βAPP_sw_ + NPD1; *R *p*<0.01 vs. βAPP_sw_ + rosiglitazone; **p<0.01 vs. βAPP_sw_ + PPARγ co-transfection.

PPARγ is a key anti-inflammatory and Aβ-lowering mediator, and several polyunsaturated fatty acids and their derivatives are ligands for PPARγ. Thus, we asked whether NPD1 influences PPARγ actions, and if this could be related to its neuroprotective bioactivity. We first tested NPD1 as a potential PPARγ activator using primary human adipocyte differentiation. PPARγ is an adipogenesis modulator, and PPARγ agonists induce adipocyte differentiation. Adipogenesis assay was used for screening potential PPARγ-active compounds. Primary human pre-adipocytes were treated with 0.1, 1 and 5 µM of NPD1 or DHA during differentiation induction (see **Figure 9**). Ligand-induced differentiation was assessed by Oil Red O staining. NPD1 led to enhancement of differentiation in the primary human pre-adipocytes, while equivalent doses of its precursor DHA displayed little adipogenic activity, suggesting that NPD1 does display PPARγ activity (**Figure 9** and Figure S2). To further evaluate the activity of NPD1, we used a cell-based PPARγ transactivation reporter assay. HNG cells co-transfected with hPPARγ-GAL4 and MH100-tk-luc were incubated with increasing concentrations (0.1, 1.0, 5.0 and 10 µM) of NPD1 or DHA for 24 h. NPD1, but not its precursor DHA, increased reporter activity in a dose-dependent manner indicating that NPD1 acts as an activator of an inducible PPARγ response (**Figure 10A**).

### The anti-amyloidogenic effect of NPD1 is PPARγ-dependent

**Figure 11:**
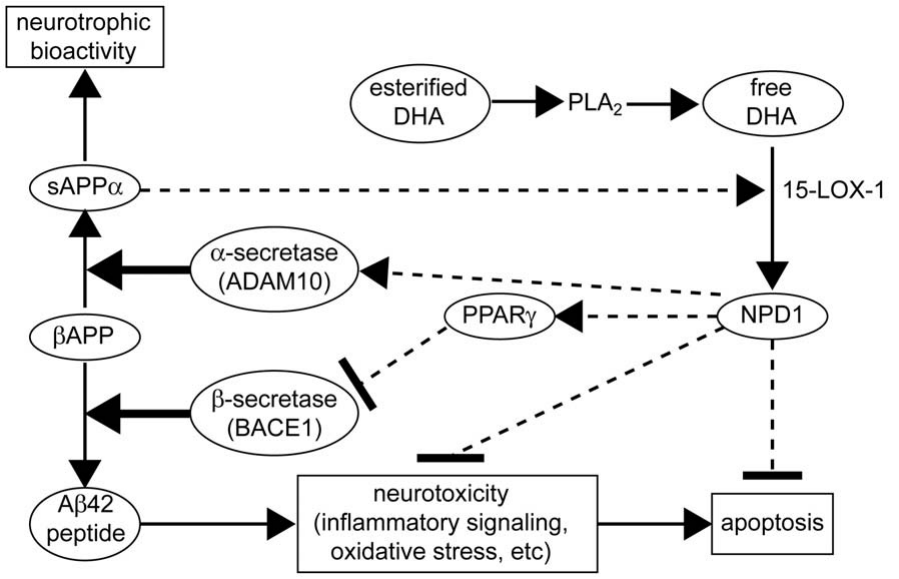
NPD1 promotes non-amyloidogenic, neurotrophic bioactivity via pleiotrophic mechanisms. Membrane esterified DHA is excised by phospholipase A2 (PLA2) to yield free DHA; in turn free DHA is 15-lipoxygenated to generate NPD1 which then enters a neuroprotective cycle. These events are mediated, in part, by inhibiting apoptosis, by blocking inflammatory signaling, by promoting cell survival and by shifting βAPP processing from an amyloidogenic into a neurotrophic, non-amyloidogenic pathway. BACE1 activity is suppressed and α-secretase (ADAM10) activity is stimulated, thus down-regulating Aβ42 peptide release from membranes. Augmentation of BACE1 and ADAM10 by NPD1 may be mediated via other neuromolecular factors. We note that the ADAM10 cleavage product sAPPα further induces the conversion of free DHA into NPD1, thus constituting a positive, neurotrophic feedback loop.

We further examined whether PPARγ is involved in the regulation by NPD1 of βAPP processing. First, we studied the effect of PPARγ on Aβ42 peptide production in HNG cells over-expressing βAPP_sw_ by either transiently-transfecting PPARγ cDNA or using the PPARγ agonist, rosiglitazone. The efficiency of transfection and subcellular localization of both proteins were monitored by immunofluorescence and Western blotting. The majority of PPARγ expression was found to be associated with the HNG cell nuclei (**Figure 10B,C**). In both PPARγ-transfected and PPARγ agonist-treated HNG cells, we observed a decrease in the amount of secreted total Aβ and Aβ42. The decrease was comparable to that conferred by NPD1 treatment. To determine whether PPARγ is required in this action, HNG cells were also incubated with the PPARγ antagonist, GW9662. GW9662 reversed the Aβ peptide reduction in NPD1-treated cells and in PPARγ over-expression or PPARγ agonist-treated cells as well (**Figure 11A**). These results suggest that PPARγ is required for NPD1's regulation of Aβ release. To further define the action of PPARγ on βAPP processing and its implication in the anti-amyloidogenic effect of NPD1, we analyzed the levels of βAPP fragments using the above treatments. Similar to NPD1-induced reduction in sAPPβ and CTFβ, in cells over-expressing PPARγ or treated with PPARγ agonist, these two β-secretase cleavage products were substantially down-regulated (**Figure 11B,D,E**). Just as in the case of Aβ peptide release, this down-regulatory effect was reversed by the addition of PPARγ antagonist in all relevant treatment groups (**Figure 11B,D,E**). Note that GW9662 alone caused no changes in either sAPPβ_sw_ or CTFβ (**Figure 11C**). In contrast, unlike NPD1, PPARγ overexpression or PPARγ agonist did not modify the levels of sAPPα or CTFα. Nor did the PPARγ antagonist abolish the NPD1-induced increase in these fragments (**Figure 11B,D,E**). Meanwhile, no changes in holo-βAPP by PPARγ were observed (**Figure 11B**). These data suggest that PPARγ is involved in NPD1's regulation via the β-secretase pathway but not via the α-secretase pathway. We next examined the levels of ADAM10 and BACE1, the putative α- and β-secretase that are actively involved in NPD1's modulation of APP processing. In agreement with the alterations in levels of βAPP fragments, PPARγ activation reduced the steady-state level of BACE1 expression but did not affect ADAM10. PPARγ antagonism abolished the NPD1-induced decrease in BACE1 but was not able to reverse the increase in mature ADAM10 level (**Figure 11B,F**).

## Discussion

DHA partially counteracts cognitive decline in the elderly [11]. Moreover, omega-3 essential fatty acid-rich diets are associated with a trend in reduced risk for MCI and with MCI conversion to AD, whereas DHA has been shown to be beneficial in transgenic AD models [8], [10], [11], [16], [50]. The 15-lipoxygenase-1- (15-LOX-1) DHA-derived NPD1 displays neuroprotective bioactivity in brain and retinal cells against various insults, including oxidative injury, ischemia-reperfusion and inflammation [9], [17], [18], [51]–[53]. Both AD brain [9] and the 3xTg-AD mouse exhibit reductions in DHA and NPD1 (**Figure 1**). In this study we further characterized the anti-inflammatory and anti-apoptotic activity of NPD1 in co-cultures of HNG cells stressed with Aβ42 oligomer, and studied the NPD1-mediated modulation of α- and β-secretase activity that resulted in reduced shedding of Aβ42.

AD is marked by synaptic damage, neuronal atrophy and cell death in the hippocampus and entorhinal cortex [4], [54]–[56]. Neurotoxicity induced by Aβ42 aggregates appears to drive microglial-mediated neuroinflammatory responses and apoptosis [3], [4], [50], [57]. Oxidative stress, calcium overload, mitochondrial dysfunction and membrane impairments, along with activation of caspases and cell death are associated with Aβ42 up-regulation [55]. We found that NPD1 induces HNG cell survival after Aβ42-oligomer-mediated stress and reduced Aβ42-triggered apoptosis. NPD1 attenuated caspase-3 activation and decreased compacted nuclei and fragmented DNA [18], [19] (**Figure 3**). These observations are in agreement with the NPD1-mediated up-regulation of anti-apoptotic Bcl-2, Bcl-xl and Bfl-1 expression and the decrease in the pro-apoptotic expression of Bax, Bad and Bik [9], [18].

Neuroinflammatory neurodegeneration associated with Aβ42 is an important contributory event to AD neuropathology [54], [56]. In these experiments primary HNG cells were used, as human primary neurons do not survive well in the absence of glial cells [9], [29] (**Figure 2**). While we cannot exclude the possibility that glial cells are providing some neuroprotective ‘shielding’, both neuronal and glial cells release cytokines when exposed to Aβ42 that, in turn, activate more microglia and astrocytes that reinforce pathogenic signaling. NPD1 is anti-inflammatory and promotes inflammatory resolution [17], [18], [37], [53]. In HNG cell models of Aβ42 toxicity, microarray analysis and Western blot analysis revealed down-regulation of pro-inflammatory genes (COX-2, TNF-α and B94), suggesting NPD1's anti-inflammatory bioactivity targets, in part, this gene family [9]. These effects are persistent, as shown by time-course Western blot analysis in which protein expression was examined up to 12 h after treatment by Aβ42 and NPD1.

Although counteracting Aβ42-induced neurotoxicity is a promising strategy for AD treatment, curbing excessive Aβ42 release during neurodegeneration is also desirable. DHA could lower Aβ42 load in the CNS by stimulating non-amyloidogenic βAPP processing, reducing PS1 expression, or by increasing the expression of the sortilin receptor, SorLA/LR11 [8], [21], [41], [58]. In contrast to a previous report by Green et al. [16] that suggested that Aβ peptide reductions in whole brain homogenates of 3xTg AD after dietary supplementation of DHA were the result of decreases in the steady state levels of PS1, our experiments in primary HNG cells showed no effects of NPD1 on PS1 levels, but a significant increase in ADAM10 coupled to a decrease in BACE1 (**Figure 5**). These later observations were further confirmed by both activity assays (**Figures 6** **and** **7**) and siRNA knockdown (**Figure 8**). NPD1 reduces Aβ42 levels released from HNG cells over-expressing APP_sw_ in a dose-dependent manner. Our examination of other βAPP fragments revealed after NPD1 addition, a reduction in the β-secretase products sAPPβ_sw_ and CTFβ occurred, along with an increase in α-secretase products sAPPα and CTFα, while levels of βAPP expression remained unchanged in response to NPD1. Hence these abundance- and activity-based assays indicate a shift by NPD1 in βAPP processing from the amyloidogenic to non-amyloidogenic pathway. Previously sAPPα has been found to promote NPD1 biosynthesis from DHA [9], while in the present study NPD1 works to stimulate sAPPα secretion, creating positive feedback and neurotrophic reinforcement. Secreted sAPPα's beneficial effects include enhanced learning, memory and neurotrophic properties [6]. NPD1 further down-regulated the β-secretase BACE1 and activated ADAM10, a putative α-secretase. Our ADAM10 siRNA knockdown and BACE1 over-expression-activity experiments confirmed that ADAM10 and BACE1 are required in NPD1's regulation of βAPP. NPD1 therefore appears to function favorably in both of these competing βAPP processing events.

PPARγ activation leads to anti-inflammatory, anti-amyloidogenic actions and anti-apoptotic bioactivity, as does NPD1. Some fatty acids are natural ligands for PPARγ, which have a predilection for binding polyunsaturated fatty acids [59]–[61]. Our hypothesis that NPD1 is a PPARγ activator was confirmed by results from both human adipogenesis and cell-based-transactivation assay (**Figures 9** **and** **10**). NPD1 may activate PPARγ via direct binding or other interactive mechanisms [33], [62]. Analysis of βAPP-derived fragments revealed that PPARγ does play a role in the NPD1-mediated suppression of Aβ production. Over-expressing PPARγ or incubation with a PPARγ agonist led to reductions in Aβ, sAPPβ and CTFβ similar to that with NPD1 treatment, while a PPARγ antagonist abrogated these reductions. Activation of PPARγ signaling is further confirmed by the observation that PPARγ activity decreased BACE1 levels, and a PPARγ antagonist overturned this decrease. Thus, the anti-amyloidogenic bioactivity of NPD1 is associated with activation of the PPARγ and the subsequent BACE1 down-regulation. The difference between the bioactivity of NPD1 concentrations for anti-apoptotic and anti-amyloidogenic activities (50 nM vs. 500 nM) may be due to the different cell models used (i.e., Aβ-peptide stressed vs. βAPP_sw_-over-expressing HNG cells) and/or related mechanisms.

Although Aβ-lowering effects of PPARγ have been reported, the molecular mechanism of this action remains unclear. Induction of βAPP ubiquitination, which leads to enhanced βAPP degradation and reduced Aβ peptide secretion, has been suggested [60]. Alternatively, Aβ clearance might be involved, or regulation by PPARγ may be due to enhancement of insulin sensitivity and increases in brain insulin degrading enzyme [59]. Our results suggest that decreases in BACE1 may be the cause for Aβ reduction [27], [63]. A reason for these conflicting reports may be that cell models and culture conditions used varied; in our study, we used HNG cells transiently over-expressing βAPP_sw_ while previous reports employed cell lines using stable βAPP expression. Similar to the model of Sastre et al. [63], our cells underwent increases in αβ overproduction. Excessive Aβ causes inflammatory responses in both neuronal and glial cells [27]. Since inflammatory signaling plays a role in AD pathogenesis, we believe HNG cell cultures are a valuable model for Aβ42 -mediated cellular actions. The fact that comparable results of our study were obtained at a much lower drug concentration (0.5 µM of rosiglitazone vs. 10–30 µM in previous reports) (**Figure 10**) underscores the highly sensitive nature of HNG cells after βAPP transfection. It is still possible that PPARγ may repress BACE1 by antagonizing activities of other transcription factors that promote BACE1 expression, such as STAT1, NF-κB and AP1 [64]. It is noteworthy that BACE1 expression in HNG cells was increased after βAPP over-expression. The fact that PPARγ did not affect the levels of sAPPα and CTFα besides PPARγ antagonist being unable to reverse NPD1-elicited increase in these fragments, clearly show that PPARγ is not essential for NPD1's regulation on the non-amyloidogenic pathway. Further analysis of ADAM10 showed no change occurring in ADAM10 following PPARγ activation, nor did PPARγ antagonists affect NPD1-enhanced expression of mature ADAM10. Therefore, modulation by NPD1 of α-secretase and βAPP processing are independent of PPARγ. ADAM10 is synthesized as an inactive zymogene and is processed to its mature form by cleavage of the pro-domain by pro-protein convertases (PPCs), such as furin and PC7 [65]. Other evidence also demonstrated that protein kinase C (PKC) and mitogen-activated protein (MAP) kinase, particularly extracellular signal-regulated kinases (ERK1/2), are involved in regulation of α-secretase activity [62], [66], [67]. No cross-talk between the PCs and PKC or MAP kinases has been reported. Since in our study only the mature ADAM10 was increased, it is likely that the PPCs are implicated in NPD1 actions.

PPARγ antagonist GW9662 also failed to reverse the anti-apoptotic effect of NPD1, indicating that PPARγ is not implicated in NPD1 anti-apoptotic bioactivity (**Figure 10**). NPD1 attained this neuroprotection at a concentration of 50 nM, at which its PPARγ activity is far from physiologically relevant in the *in vitro* system. Other mechanisms have been proposed to explain DHA's anti-apoptotic and anti-inflammatory effects, including maintenance of plasma membrane integrity, activation of Akt signaling [68], and conversion into other derivatives [23], [50]. These findings also provide clues for NPD1's potential targets. NPD1 inhibits NF-κB activation and COX-2 expression in brain ischemia-reperfusion [17], while Aβ peptide-induced apoptosis is associated with ERK and p38 MAPK-NF-κB mediated COX-2 up-regulation [44]. Neuroprotection mediated by NPD1 may further involve components of signaling pathways upstream of NF-κB activation and DNA-binding [9].

Our results provide compelling evidence that NPD1 is endowed with strong anti-inflammatory, anti-amyloidogenic, and anti-apoptotic bioactivities in HNG cells upon exposure to Aβ42 oligomers, or in HNG cells over-expressing βAPP_sw_. These results suggest that NPD1's anti-amyloidogenic effects are mediated in part through activation of the PPARγ receptor, while NPD1's stimulation of non-amyloidogenic pathways is PPARγ-independent. Suggested sites of NPD1 actions are schematically presented in **Figure 11**. NPD1 stimulation of ADAM10 coupled to suppression of BACE1-mediated Aβ42 secretion clearly warrants further study, as these dual secretase-mediated pathways may provide effective combinatorial or multi-target approaches in the clinical management of the AD process.

## Supporting Information

Figure S1 **Characterization of Aβ peptide preparations using LMW-Western analysis.** (**A**) Lanes 1 and 2 represent duplicate Aβ peptide preparations prepared and analyzed by one of the authors (WJL), and (**B**) Aβ peptide preparations prepared and analyzed completely independently by another one of the authors (YZ); both Aβ peptide preparations were prepared and analyzed according to the HFIP (hexafluoroisopropanol; 1,1,1,3,3,3-hexafluoro-2-propanol) preparative and gel analytical methods described by Stine et al., (J Biol Chem. 278:11612-22,2003). No higher order Aβ fibrils are evident in either (**A**) or (**B**). Panel (**C**) shows relative toxicity of monomer, fibril and oligomer preps shown in (**B**) as analyzed using MTT [3-(4,5-dimethylthiazol-2-yl)-2,5-diphenyltetrazolium; Invitrogen] cell viability assay.
(TIF)

Figure S2 **Dose response of vehicle (control), NPD1 and DHA (at 0.1, 0.5, 1.0, 5.0 and 10.0 μM) on PPARγ activity - effects on PPARγ activity using luciferase reporter fold change over controls.** Experimental conditions are further described in the text.
(TIF)
