# Supplementary figures and images for: Docosahexaenoic Acid-Derived Neuroprotectin D1 Induces Neuronal Survival via Secretase- and PPARγ-Mediated Mechanisms in Alzheimer's Disease Models
Source: PLoS One. 2011 Jan 5;6(1):e15816. doi: 10.1371/journal.pone.0015816 (PMC3016440; doi:10.1371/journal.pone.0015816)

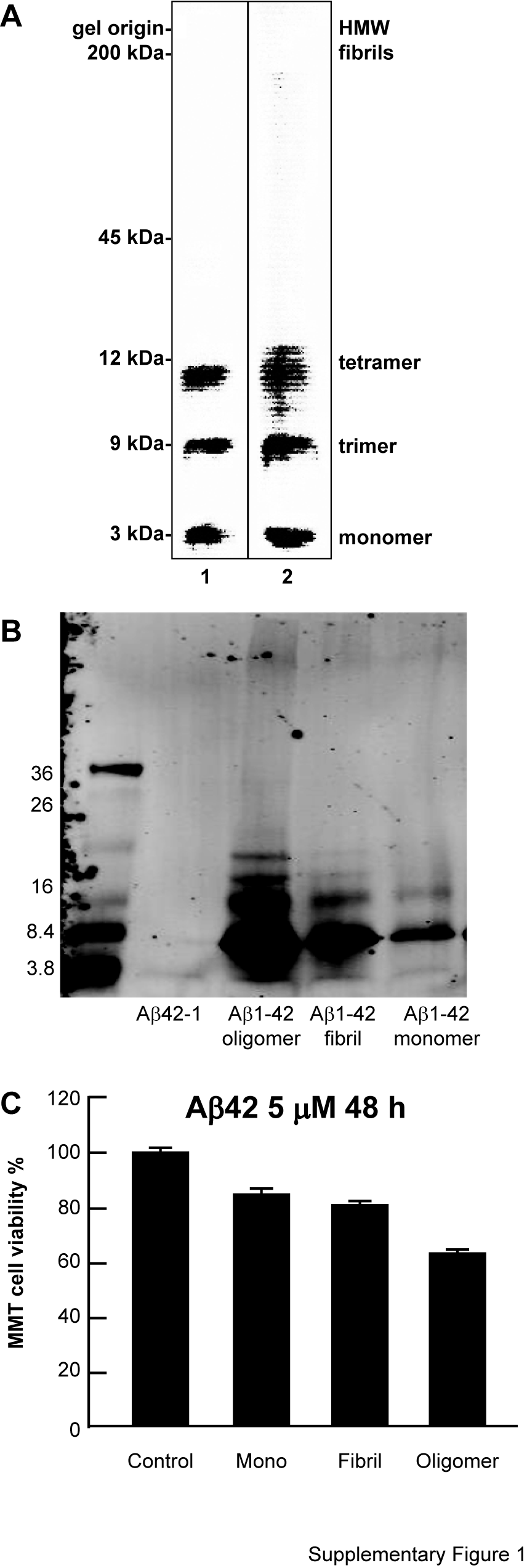

Supplement: Figure S1 — Characterization of Aβ peptide preparations using LMW-Western analysis. (A) Lanes 1 and 2 represent duplicate Aβ peptide preparations prepared and analyzed by one of the authors (WJL), and (B) Aβ peptide preparations prepared and analyzed completely independently by another one of the authors (YZ); both Aβ peptide preparations were prepared and analyzed according to the HFIP (hexafluoroisopropanol; 1,1,1,3,3,3-hexafluoro-2-propanol) preparative and gel analytical methods described by Stine et al., (J Biol Chem. 278:11612-22,2003). No higher order Aβ fibrils are evident in either (A) or (B). Panel (C) shows relative toxicity of monomer, fibril and oligomer preps shown in (B) as analyzed using MTT [3-(4,5-dimethylthiazol-2-yl)-2,5-diphenyltetrazolium; Invitrogen] cell viability assay. (TIF) [file pone.0015816.s001.tif]

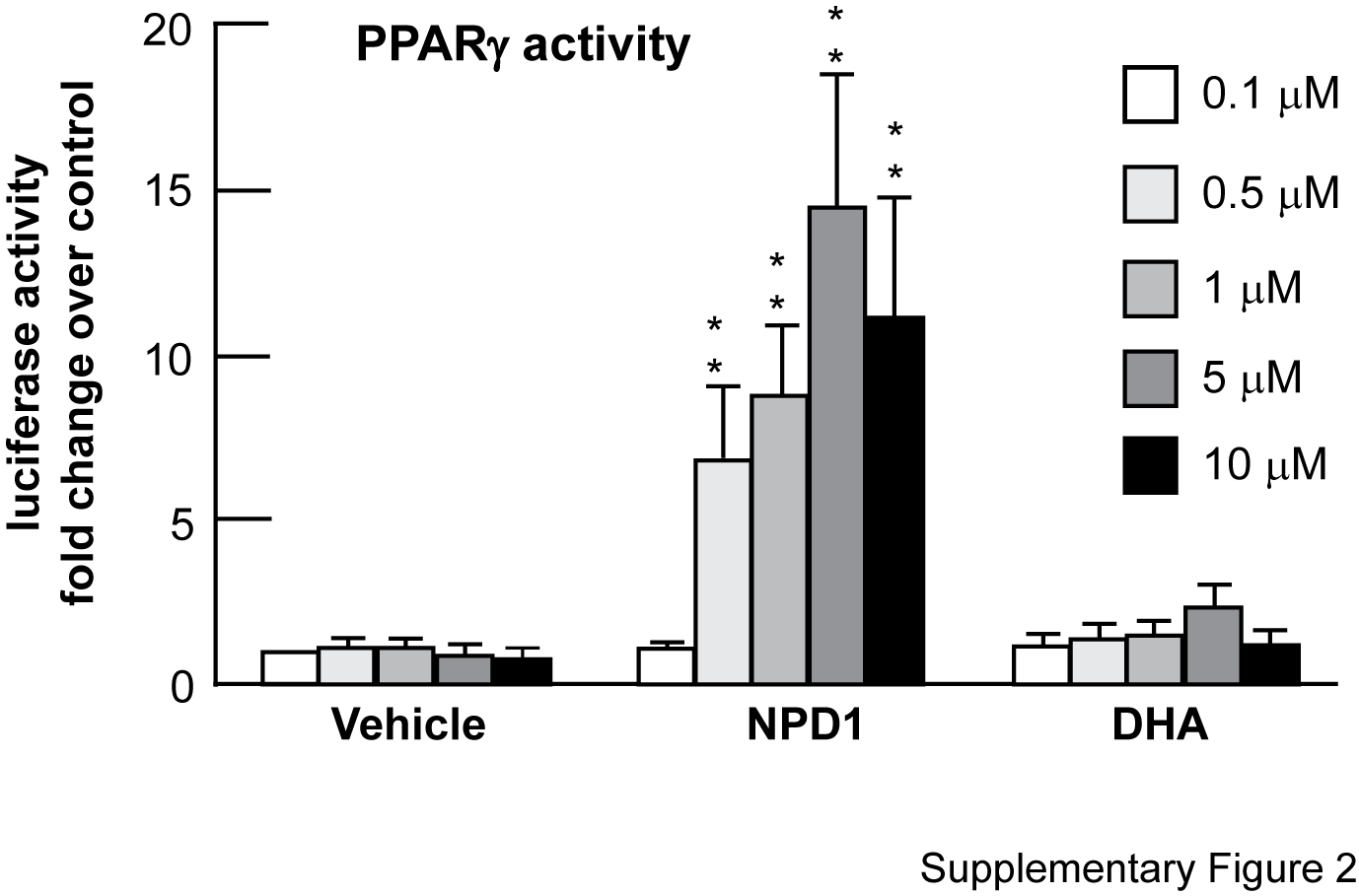

Supplement: Figure S2 — Dose response of vehicle (control), NPD1 and DHA (at 0.1, 0.5, 1.0, 5.0 and 10.0 μM) on PPARγ activity - effects on PPARγ activity using luciferase reporter fold change over controls. Experimental conditions are further described in the text. (TIF) [file pone.0015816.s002.tif]
